# Paleogenomics retraces the ancient population history of Southwestern East Asia for the past 12 000 years

**DOI:** 10.1093/nsr/nwag398

**Published:** 2026-07-02

**Authors:** Fan Bai, Xinglong Zhang, Yongxu Fu, Hongliang Lu, Fei Li, E Andrew Bennett, Shuwen Pei, Peng Cao, Qingyan Dai, Feng Liu, Xiaotian Feng, Ming Zhang, Wenjun Wang, Fajun Li, Ganyu Zhang, Wanjing Ping, Tianxiang Liu, Qiaomei Fu

**Affiliations:** Key Laboratory of Vertebrate Evolution and Human Origins of Chinese Academy of Sciences, Institute of Vertebrate Paleontology and Paleoanthropology, Chinese Academy of Sciences, Beijing 100044, China; School of Archaeology and Museology, Sichuan University, Chengdu 610207, China; Guizhou Provincial Museum, Guiyang 550081, China; Institute of Archaeology, Chinese Academy of Social Sciences, Beijing 100710, China; School of Archaeology and Museology, Sichuan University, Chengdu 610207, China; Center for Archaeological Science, Sichuan University, Chengdu 610207, China; Guizhou Provincial Institute of Cultural Relics and Archaeology, Guiyang 550003, China; Key Laboratory of Vertebrate Evolution and Human Origins of Chinese Academy of Sciences, Institute of Vertebrate Paleontology and Paleoanthropology, Chinese Academy of Sciences, Beijing 100044, China; Key Laboratory of Vertebrate Evolution and Human Origins of Chinese Academy of Sciences, Institute of Vertebrate Paleontology and Paleoanthropology, Chinese Academy of Sciences, Beijing 100044, China; Key Scientific Research Base on Paleolithic Human Evolution and Paleogenetics, Beijing 100044, China; Key Laboratory of Vertebrate Evolution and Human Origins of Chinese Academy of Sciences, Institute of Vertebrate Paleontology and Paleoanthropology, Chinese Academy of Sciences, Beijing 100044, China; Key Laboratory of Vertebrate Evolution and Human Origins of Chinese Academy of Sciences, Institute of Vertebrate Paleontology and Paleoanthropology, Chinese Academy of Sciences, Beijing 100044, China; Key Laboratory of Vertebrate Evolution and Human Origins of Chinese Academy of Sciences, Institute of Vertebrate Paleontology and Paleoanthropology, Chinese Academy of Sciences, Beijing 100044, China; Key Laboratory of Vertebrate Evolution and Human Origins of Chinese Academy of Sciences, Institute of Vertebrate Paleontology and Paleoanthropology, Chinese Academy of Sciences, Beijing 100044, China; China-Central Asia “The Belt and Road” Joint Laboratory on Human and Environment Research, Key Laboratory of Cultural Heritage Research and Conservation, School of Culture Heritage, Northwest University, Xi’an 710127, China; Key Laboratory of Vertebrate Evolution and Human Origins of Chinese Academy of Sciences, Institute of Vertebrate Paleontology and Paleoanthropology, Chinese Academy of Sciences, Beijing 100044, China; Science and Technology Archaeology, National Centre for Archaeology, Beijing 100013, China; Department of Anthropology, School of Sociology and Anthropology, Sun Yat-Sen University, Guangzhou 510275, China; Key Laboratory of Vertebrate Evolution and Human Origins of Chinese Academy of Sciences, Institute of Vertebrate Paleontology and Paleoanthropology, Chinese Academy of Sciences, Beijing 100044, China; College of Earth and Planetary Sciences, University of the Chinese Academy of Sciences, Beijing 100049, China; Key Laboratory of Vertebrate Evolution and Human Origins of Chinese Academy of Sciences, Institute of Vertebrate Paleontology and Paleoanthropology, Chinese Academy of Sciences, Beijing 100044, China; Key Laboratory of Vertebrate Evolution and Human Origins of Chinese Academy of Sciences, Institute of Vertebrate Paleontology and Paleoanthropology, Chinese Academy of Sciences, Beijing 100044, China; College of Earth and Planetary Sciences, University of the Chinese Academy of Sciences, Beijing 100049, China; Key Laboratory of Vertebrate Evolution and Human Origins of Chinese Academy of Sciences, Institute of Vertebrate Paleontology and Paleoanthropology, Chinese Academy of Sciences, Beijing 100044, China; College of Earth and Planetary Sciences, University of the Chinese Academy of Sciences, Beijing 100049, China

**Keywords:** southwestern East Asia, Yunnan–Guizhou plateau, Guizhou province, ancient DNA, Paleolithic to Neolithic transition, basal East Asian ancestry

## Abstract

Southwestern East Asia, a key intersection linking southern East Asia, Southeast Asia, and the Tibetan Plateau, is a crucial area for the study of early human migrations. However, ancient DNA studies in the eastern part of the region, particularly in Guizhou, are still lacking. We present the first genomic study of Guizhou populations from a time period spanning 12 000 years, including five Guizhou individuals older than 10 000 years. Combined with published Guangxi and Yunnan samples, we reveal a complex genetic history of human populations in southwestern East Asia from the Paleolithic to the Early Neolithic periods. Paleolithic populations were marked by heterogeneous distribution patterns of the recently described basal East Asian Xingyi_EN ancestry admixed with ancestry present in northern East Asia. By the Bronze Age, this enigmatic basal ancestry was no longer detectable in the region. Beginning 1200 years ago, genetic interactions between Guizhou and Guangxi populations intensified following language-specific patterns. Overall, our results clarify the complex population dynamics of southwestern East Asia over the past 12 000 years.

## INTRODUCTION

Southwestern East Asia encompasses the present-day Chinese provinces of Yunnan, Guizhou, and Guangxi. As a crucial corridor linking lowland East Asia, the Tibetan Plateau, and Southeast Asia, it has played a pivotal role in the migration and interaction of early Asian populations [1–4]. Recent genomic studies described a 7000-year-old Xingyi individual from central Yunnan who belonged to a ‘lost’ deeply-branching basal East Asian lineage, distinct from that of the 40 000–33 000-year-old Tianyuan ancestry of northern East Asia, and the 8000–4000-year-old Hoabinhian culture-associated ancestry in Southeast Asia [1,5–7]. All of these deep ancestries were found to have diverged prior to the differentiation of East Asian populations into southern and northern ancestries [8–10]. These studies underscored the critical importance of ancient genomic research in Southwestern East Asia for uncovering early branches of Asian populations. However, there remains a significant gap in the availability of genetic data from the Paleolithic and Early Neolithic periods in the eastern part of this region, particularly in present-day Guizhou Province.

The transition from the Paleolithic to the Neolithic period marks a pivotal turning point in the development of ancient human societies, as the introduction of new subsistence practices often accompanied population migrations and displacements [6, 11–15]. The maintenance of genetic diversity, and whether the remnants of basal East Asian lineages persisted through this process, is a key issue in understanding the population history of southern East Asians. Previous studies have shown that in the western part of the region, Late Neolithic to Bronze Age Yunnan populations carried genetic components related to the ancestors of present-day Austroasiatic speakers and ancient northern East Asians, replacing the more local Xingyi-related basal East Asian ancestry [5]. Given the lack of corresponding ancient genomic data from the neighboring Guangxi region and the absence of Xingyi-related ancestry in the southeastern coastal Fujian region [16,17], the preservation of this basal East Asian lineage during this period, as well as the interactions with more recent Neolithic East Asian ancestry from both north and south, remained poorly understood. Ancient genome research focusing on Late Neolithic populations in the Guizhou region can provide crucial insights into these questions.

Present-day southwestern East Asia has one of the highest levels of ethnic diversity in East Asia, with numerous minority groups, including Kra–Dai- and Hmong–Mien-speaking populations [18]. Genetic links have been demonstrated between these language groups and populations from Guangxi dating from 1500 to 500 years ago [16]. Investigating the genetic relationships between historical minority populations in Guizhou and Guangxi will enhance our understanding of the interaction and migration histories of present-day southwestern East Asian ethnic groups.

To address these gaps in our knowledge, we have obtained samples from 36 individuals across 8 archaeological sites in Guizhou, spanning from the Paleolithic to the historical period. This study aims to offer valuable scientific insights into the genetic characteristics of Paleolithic southwestern East Asians, their population dynamics during the establishment of Neolithic cultures, and the mechanisms leading to the present-day genetic and ethnic diversity of this region.

## RESULT

**Figure 1.**
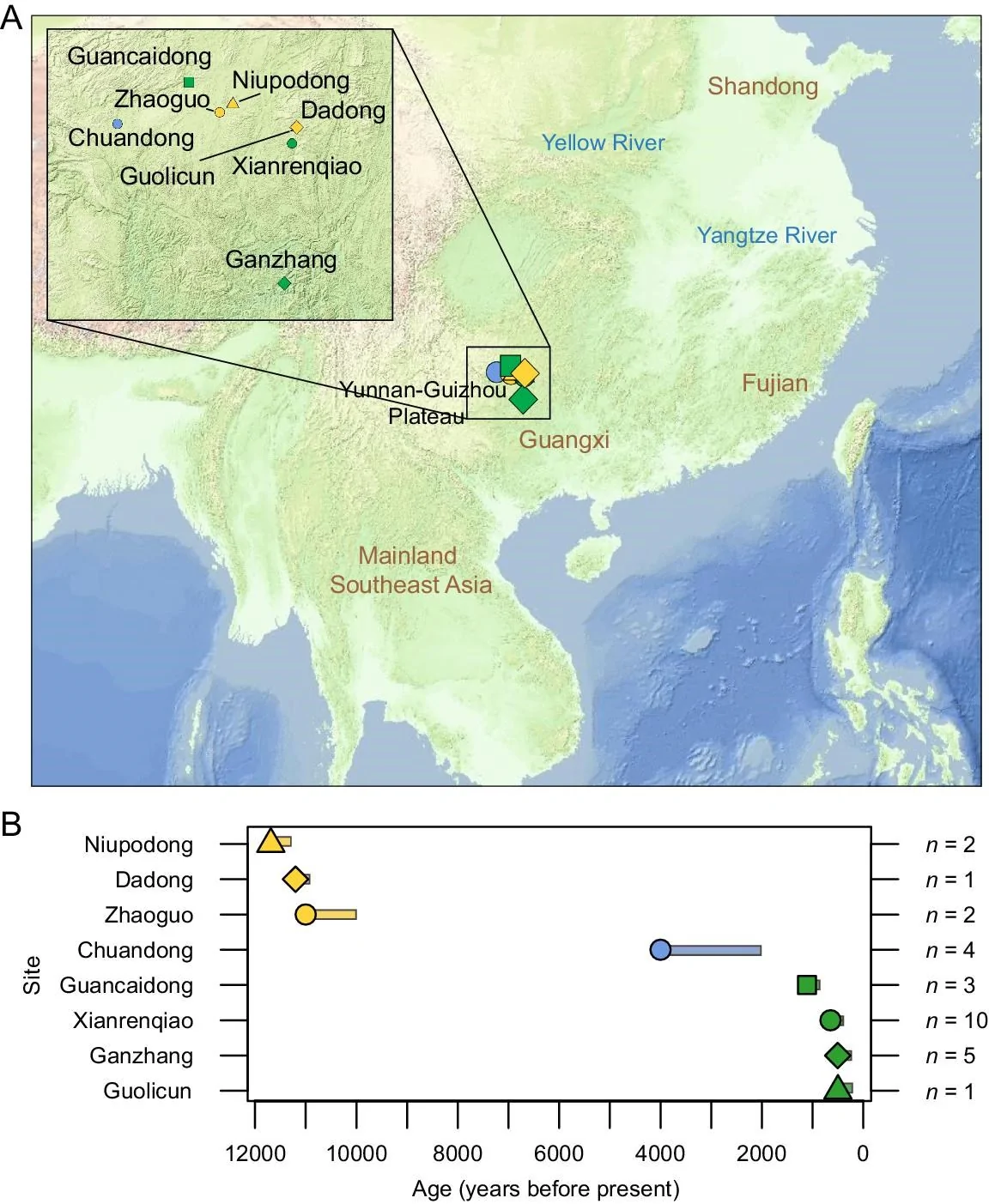
Geographical and temporal distribution of the newly sequenced ancient individuals from Guizhou. (A) Location of newly sampled sites in Guizhou. Colors distinguish the different time periods: yellow for Paleolithic (12 000–10 000 BP), blue for Neolithic-Bronze Age (4000–2000 BP), and green for historical period sites (younger than 1500 years ago). (B) Dates and number of individuals passing filters from each of the sites. The chronological bar represents either: (i) the 95% confidence interval (CI) of radiocarbon dating from one specimen at the site, (ii) the radiocarbon dating range of the specimen’s stratigraphic layer, or (iii) inferences from historical records. (See Supplementary Data section ‘Archeological information of newly sequenced samples’ for details). Detailed radiocarbon dating information is reported in Table S8. Review drawing number: GS 京(2026)1881号.

We attempted to obtain genome-wide SNP data comprising 1.2 million ancestry-informative positions from 36 individuals in the Guizhou region of southwestern East Asia. Radiocarbon dating placed the age of these remains between ∼12 000 and 300 cal BP (years before 1950 AD) (Fig. 1; Table S1, Table S8). All the results displayed typical ancient DNA damage profiles, indicative of an endogenous origin [19] (Table S1). After removing samples with an insufficient SNP number (<15 000), we estimated the modern mitochondrial contamination levels for all samples and X-chromosome contamination rates for males. For any sample with 5% or more contamination, only the DNA fragments with an ancient DNA damage signal were used for analysis [20,21]. If the contamination estimate after filtering was still ≥5%, or the remaining SNP number was <15 000, the sample was removed. We then assessed the degree of kinship between each pair of the remaining samples, and found two closely related individuals (second-degree kinship) and two genetically identical (same individual) samples. For each of these pairs, we selected the sample with the highest SNP count for inclusion in the population analyses.

After the quality and kinship filtering, we kept 28 unrelated individuals having a SNP count between 17 999 and 877 796 of the 1.2 million target SNPs [22,23] (Table S1). To explore the genetic relationship of ancient Guizhou populations with surrounding East Asians, we combined the newly sequenced data with published ancient northern and southern East Asians, Southeast Asians, and neighboring Eurasian populations.

### Genetic profiles of southwestern East Asians from the Paleolithic to the Early Neolithic period

Five out of twenty-eight newly sequenced individuals passing filters date to over 10 000 years ago, covering key Paleolithic sites in Guizhou, including Zhaoguo, Niupodong, and Dadong. These new data triple the current number of Paleolithic genomes from southern East Asia older than 10 000 years (Fig. 1; Table S1, Table S8). We merged these with the published Paleolithic to Early Neolithic populations in southwestern East Asia to create a comprehensive genomic dataset.

**Figure 2.**
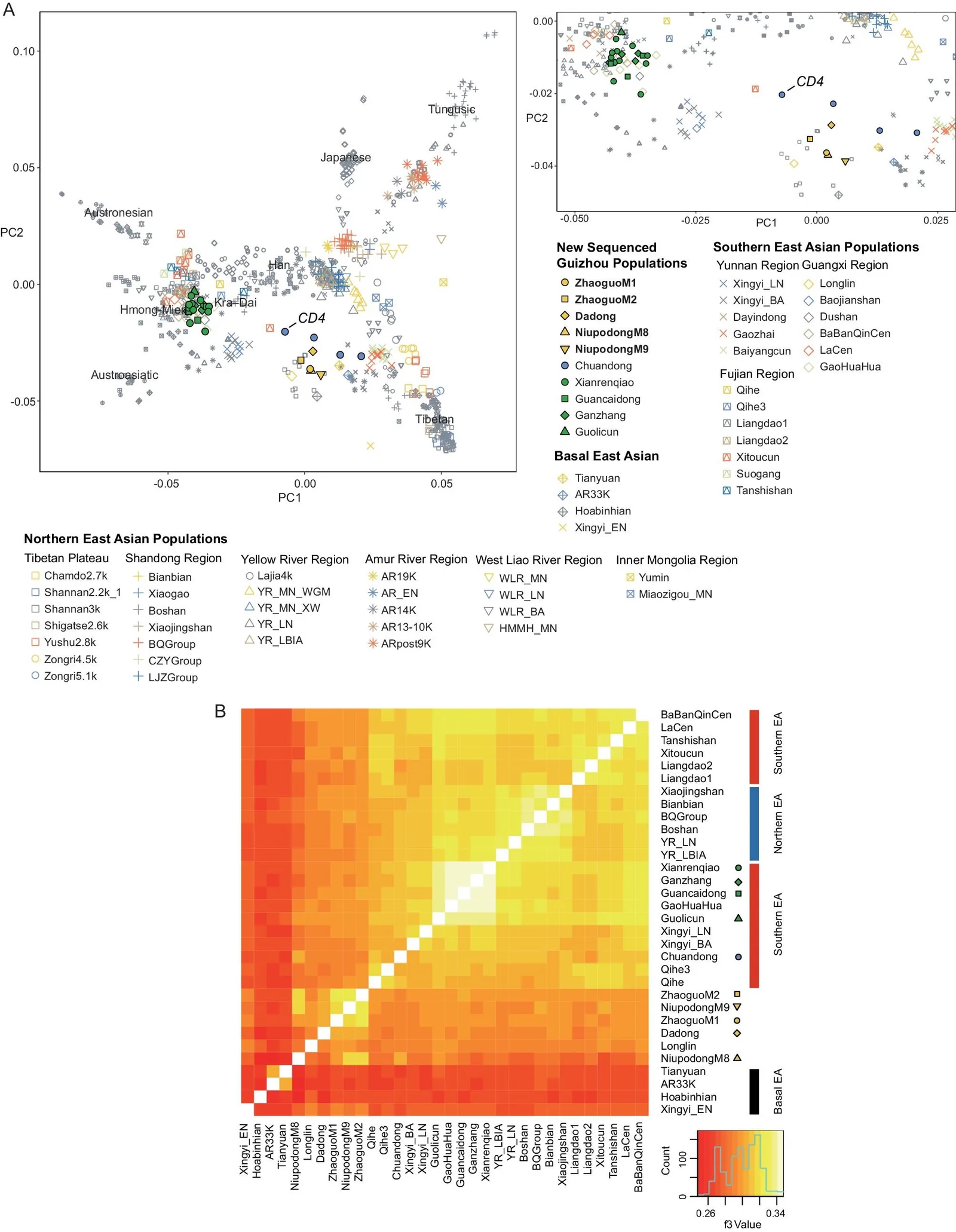
Genetic profile of newly sequenced ancient individuals from Guizhou. (A) PCA of ancient individuals projected onto the PCA space calculated using present-day Asian populations. The top-right plot shows a zoomed-in view of ancient Guizhou individuals. Yellow points are Paleolithic Guizhou samples, purple/blue points are Late Neolithic/Bronze Age Guizhou samples, and green points are historical Guizhou samples. The populations used to represent basal East Asian, Neolithic northern, and Neolithic southern East Asian groups are labeled in the legend. Paleolithic East Asian populations are shown in bold. The Chuandong individual with the highest SNP number (*CD4*; see Table S1) is specifically labeled in the plot. (B) Outgroup-*f3* heatmap, including ancient Guizhou, basal East Asians, and post-Paleolithic northern and southern East Asians. The sample ID, age information, and detailed geographic location of published ancient samples used in analysis can be found in Table S11.

To explore the genetic characteristics of these early southwestern East Asian populations, we projected them onto the principal component space calculated using present-day Asian populations. All five Paleolithic Guizhou individuals formed a discrete cluster in principal component analysis (PCA) analysis, and outgroup f3 statistics, which measures the amount of shared genetic drift between two test populations, demonstrated they shared similar ancestry (Fig. 2). To test this further and explore how deeply diverged these Paleolithic Guizhou samples were, we compared them with Neolithic and basal East Asian lineages using D-statistics. Compared to the basal East Asian ancestries of Tianyuan and Hoabinhian (represented by La368), the Paleolithic Guizhou samples shared more alleles with Neolithic East Asians (D(*Neolithic East Asians, Tianyuan/AR33K/Hoabinhian; ZhaoguoM1/ZhaoguoM2/NiupodongM9/Dadong, Mbuti*), *Z* = 2.1–11.4, Table S3). However, compared with the recently described basal East Asian ancestry Xingyi_EN, localized in the surrounding region, the Paleolithic Guizhou individuals, except for Dadong, were equally distant genetically from this deeply branching basal ancestry as they were from Neolithic East Asians in nearly all cases (Table S3). Overall, Paleolithic Guizhou individuals showed no significant patterns of genetic affinity for either northern or southern Neolithic East Asian ancestry (Table S3), and the Paleolithic Guizhou individuals, represented by the better covered ZhaoguoM1 and ZhaoguoM2, shared more genetic drift with Xingyi_EN than nearly all other ancient East Asians, except Zongri5.1k and Longlin (*D(ZhaoguoM1/ZhaoguoM2, ancient East Asians except Zongri5.1k and Longlin; Xingyi_EN, Mbuti), Z* = 2.3–9.5, Table S9). This pattern was expected as both Zongri5.1k and Longlin were shown to contain Xingyi_EN-related ancestry [5]. These results suggest the Paleolithic Guizhou samples contained a degree of Xingyi_EN ancestry that diverged earlier than the separation of northern and southern East Asian populations.

**Figure 3.**
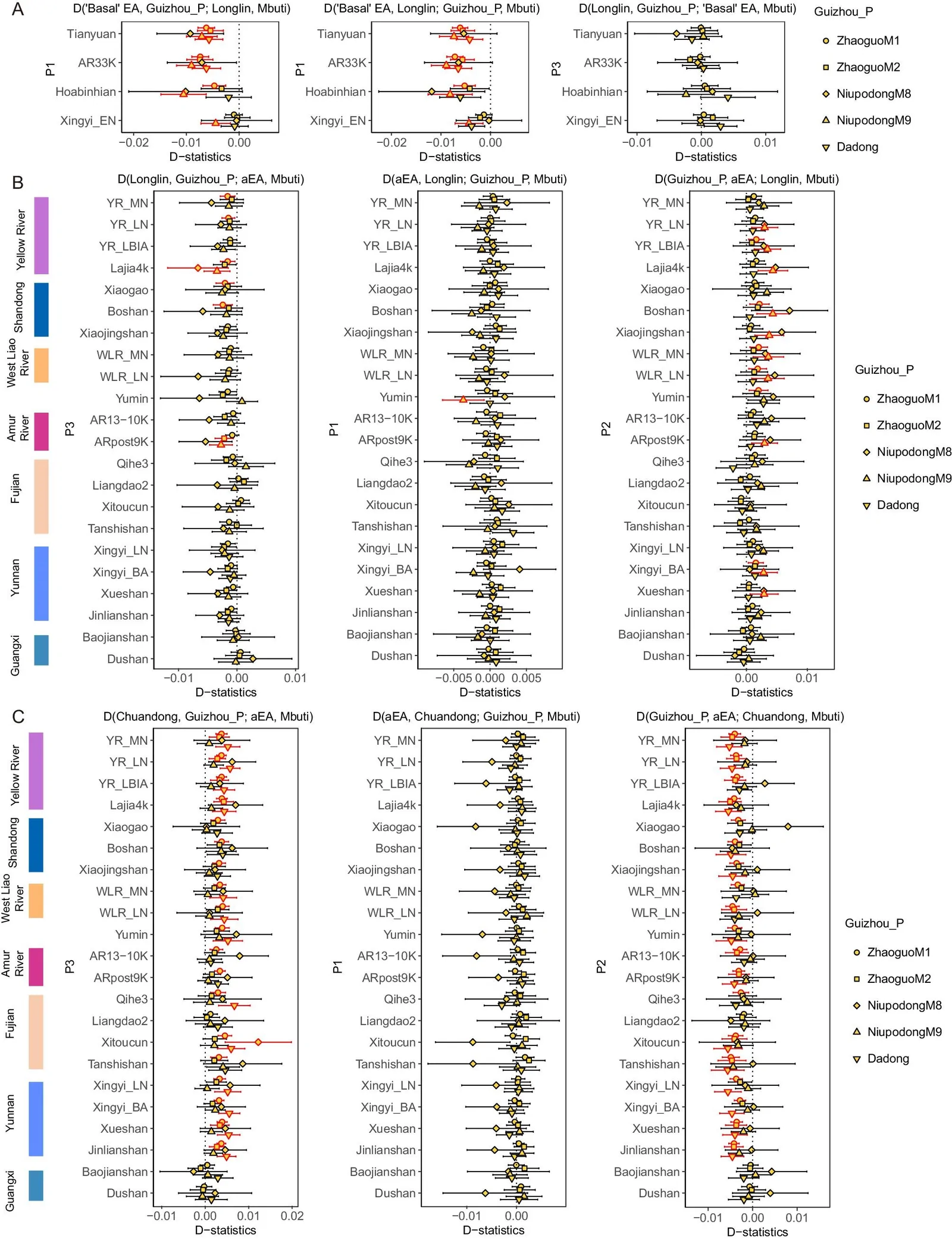
The genetic characteristics of Paleolithic and Neolithic Guizhou populations. (A) D-statistics depicting the relationship among basal East Asians (on the *Y* axis), the Paleolithic Guizhou populations (‘Guizhou_P’, symbols), and Longlin. (B) Comparing Longlin and the Paleolithic Guizhou with northern and southern East Asian ancestries. (C) Comparing the Paleolithic Guizhou and the Late Neolithic to Bronze Age Chuandong with northern and southern East Asian ancestries. For the plots a–c, the error bars represent 2 times the standard error, and the D-statistics with |*Z*| > 2.5 are highlighted with a red outline and error bar. The sample ID, age information, and detailed geographic location of published ancient samples used in analysis can be found in Table S11.

The 11 000-year-old Longlin from Guangxi is another Paleolithic individual carrying deeply diverged East Asian ancestry, who was found to be a mixture of basal East Asian Xingyi_EN-related ancestry and Neolithic southern East Asian ancestry [5]. Longlin also shares more alleles with Neolithic East Asians than with basal Tianyuan and Hoabinhian individuals, but is equally close to basal Xingyi_EN and Neolithic East Asians [16], similar to the Paleolithic Guizhou samples. Compared with the basal East Asian ancestries of Tianyuan and Hoabinhian, both Longlin and the Paleolithic Guizhou individuals were more similar genetically to each other, and could not be differentiated with Xingyi_EN ancestry (Fig. 3A). The maximum likelihood phylogenetic tree also showed the ZhaoguoM1 individual acted as an outgroup to Neolithic northern and southern East Asians, similarly to Longlin and Xingyi_EN (Fig. S4). These results suggest the Paleolithic Guizhou samples share the same basal East Asian Xingyi_EN-related ancestry found in Longlin.

**Figure 4.**
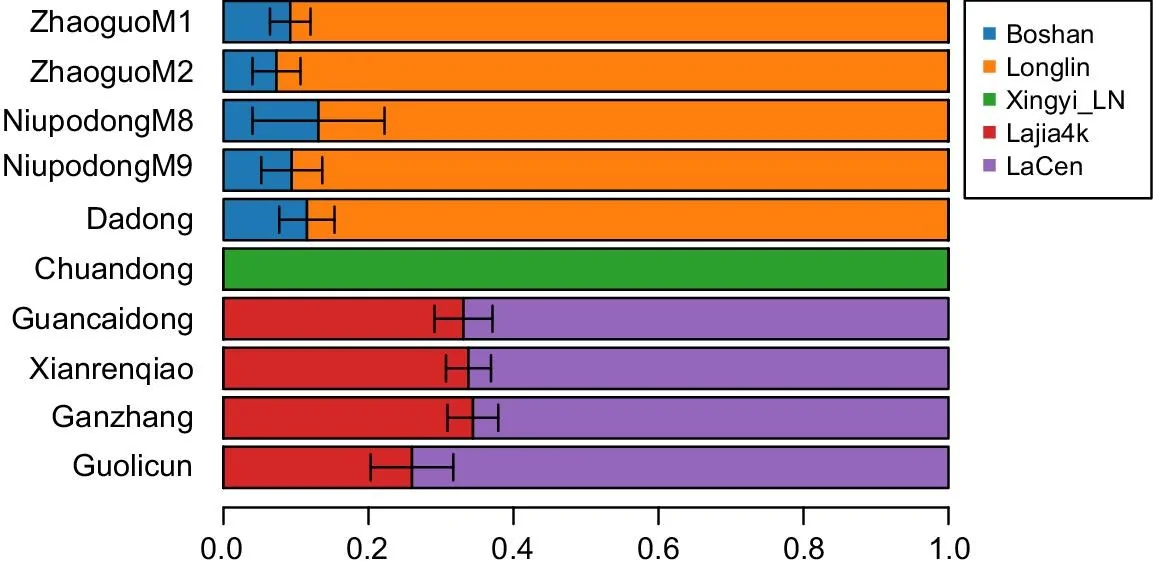
Representative admixture models from newly sequenced Guizhou sites. Presented models were selected based on convergence across multiple analyses, including admixture models inferred from Treemix and qpGraph and the patterns of observed D-statistics. The full set of feasible models, including all non-rejected one-way models, is available in Table S5–S7. Colors represent different representative ancestry sources as indicated in the legend, which are as follows: Longlin, Paleolithic southern East Asian populations with Xingyi_EN related ancestry; Boshan, broad Early Neolithic northern East Asian ancestry not restricted to the Shandong region; Xingyi_LN, Late Neolithic ancestry found in Yunnan; Lajia4k, Late Neolithic Yellow River ancestry; and LaCen, Historical Guangxi ancestry. Other feasible models for each population are listed in Table S5–S7. Details about the qpAdm modeling process and model selection are described in the Supplementary Data section, ‘Admixture modeling with qpAdm’. Error bars represent ±1 SE. The sample ID, age information, and detailed geographic location of published ancient samples used in the analysis can be found in Table S11.

To further assess the genetic differences between the Paleolithic Guizhou samples and Longlin relative to Neolithic northern and southern East Asians, D-statistics results show that the Paleolithic Guizhou samples, especially the 11 000-year-old ZhaoguoM1, which has the highest SNP count, had significantly more northern East Asian ancestry similar to that found in Neolithic period populations from both coastal Shandong and the inland Yellow River region than the Longlin individual (Fig. 3B). Although the 9000–6000-year-old Guangxi populations from Dushan and Baojianshan were admixed with Longlin ancestry and southern coastal Fujian ancestry, and Southeast Asian Hoabinhian-related ancestry, respectively, we did not find significant signals of either Fujian- or Hoabinhian-related ancestry in the Paleolithic Guizhou samples (Fig. 3A–B). Admixture graph analysis also showed the ZhaoguoM1 individual could be modeled as a mixture between Xingyi_EN-related ancestry and an ancient broad northern East Asian ancestry, which was absent in the Longlin individual (Figs S5 and S6). The northern East Asian influence on ZhaoguoM1 was also supported by the maximum likelihood phylogenetic tree (Fig. S4). If we included Longlin as a source, ZhaoguoM1 could be modeled as Longlin with additional ∼11.5%–13.4% northern East Asian (for example, Boshan or Bianbian) ancestry (Fig. 4, Table S5).

Collectively, our results reveal a complex genetic landscape in Paleolithic southwestern East Asia before 10 000 years ago, and that the ‘lost’ basal East Asian ancestry represented by the Xingyi_EN individuals in central Yunnan was widely distributed in this region. During this period, the Paleolithic populations in Guizhou and Guangxi were mixtures of Xingyi_EN-related basal East Asian and Neolithic northern or southern East Asian ancestry (See Fig. 2A for the populations used to represent basal East Asian, Neolithic northern, and Neolithic southern East Asian groups), among which the Guizhou samples had a higher proportion of northern East Asian ancestry, and the Guangxi samples had more southern East Asian ancestry. Following this period, the ancestry of the coastal Fujian populations and Southeast Asia Hoabinhian hunter-gatherers expanded into the Guangxi region [16], but was not found further inland in Guizhou.

### Basal East Asian ancestry disappears from southwestern East Asia by the Bronze Age

To examine whether the basal East Asian Xingyi_EN ancestry identified in Guizhou persisted into the Late Neolithic and Bronze Age periods, we sequenced four individuals excavated from layers dating to 4000–2000 BP of the Chuandong site. These samples bridge the temporal gap existing between the Late Neolithic and the Historical era in southwestern East Asia outside of Yunnan. In the PCA plot, the four Chuandong individuals clustered closer to Neolithic northern East Asian populations, such as those in Shandong or the Yellow River region, than to the Paleolithic Guizhou samples (Fig. 2A). Since three out of four Chuandong individuals had SNP counts <25 000 (Table S1), all Chuandong samples were merged into a single group to increase statistical power.

To explore the presence of basal East Asian ancestries in the Bronze Age Guizhou region, we calculated allele sharing between Xingyi_EN and Chuandong using D-statistics. We found the Bronze Age Chuandong individuals shared no more alleles with Xingyi_EN than other ancient East Asians (Table S9), a pattern that was also consistent for later Historical Guizhou populations. To further test whether Xingyi_EN ancestry contributed to the Bronze Age southwestern East Asians, we designated the Chuandong population, as well as other Late Neolithic to Bronze Age populations from Yunnan and Fujian, as target groups in qpAdm modeling and assessed whether the Xingyi_EN population could serve as an ancestral source for these target populations. The results showed that only a small fraction of basal East Asian Xingyi_EN-related ancestry was present in the ∼2000-year-old Duizi (∼10.8%) populations from western Yunnan, and was absent in the other tested populations, including Chuandong (Table S6). Since Xingyi_EN could not be modeled as a feasible source for later Bronze Age Guizhou or Yunnan populations in qpAdm analysis, we propose that Xingyi_EN ancestry had disappeared from known southwestern East Asian populations by this period.

The Chuandong population could be one-way modeled by Xingyi_LN ancestry (Fig. 4, Table S6). Xingyi_LN ancestry, first identified in Late Neolithic Yunnan, is a regional lineage with no continuity with Xingyi_EN, and distinct from previously described northern and southern East Asian ancestry clines. In the Yunnan region, Xingyi_LN replaced the earlier local Xingyi_EN-related basal East Asian ancestry by the Late Neolithic, and is proposed to be related to the ancestors of present-day Austroasiatic speakers [5]. Based on the qpAdm results, and assuming the archeological context of the Chuandong individuals between 4000 and 2000 BP to be broadly reliable, we argue that a similar ancestry replacement also occurred in the Guizhou region around or prior to the onset of the Bronze Age (ca. 3100–3000 BP in this region [24]). To further test this hypothesis, we evaluated the genetic differences between Chuandong and the Paleolithic Guizhou populations. Overall, Chuandong shared more alleles with Neolithic East Asian populations outside of the Guangxi region than with Paleolithic Guizhou (Fig. 3C). Moreover, no Paleolithic Guizhou affinity was detected when we compared Chuandong with other Neolithic East Asian populations (Fig. 3C) (*D(aEA, Chuandong; Guizhou_P, Mbuti), −*2.2 *< Z <* 2.8). All these results support a replacement of the earlier local Xingyi_EN-related basal East Asian ancestry in Guizhou that had occurred before 4000 years ago.

However, our current results were insufficient to determine which specific ancestral population drove these genetic changes. Although the central Yunnan Xingyi_LN population was a feasible one-way source for the Chuandong population, this and other central Yunnan populations such as Xingyi_BA, Xueshan, and Jinlianshan populations showed similar D-statistics values as Neolithic northern East Asians from the Yellow River and the Shandong region, and some Fujian populations in southern East Asia (Fig. 3C). And the genetic relationship among the Chuandong population, central Yunnan populations, and Neolithic East Asia could not be resolved by D-statistics: (*D(Xingyi_LN, aYellow River/aShandong/aFujian; Chuandong, Mbuti)*, −1.9 < *Z* < 2.4; *D(Xingyi_LN, Chuandong; aYellow River/aShandong/aFujian, Mbuti)*, −3.3 < *Z* < 1.2; *D(aYellow River/aShandong/aFujian, Chuandong; Xingyi_LN, Mbuti)*, −2.5 < *Z* < −0.1; Table S10). These results support neither a Chuandong and Xingyi_LN clade, nor Chuandong being a mixture of Xingyi_LN and Neolithic Yellow River, Shandong, or Fujian populations. Among the tested Neolithic East Asians, the western Yunnan Ladian population shared the highest amount of ancestry in the *D(Ladian, Y = Neolithic East Asian; Chuandong, Mbuti)* test (Table S10). While the western Yunnan populations were also a mixture between Central Yunnan ancestry and northern East Asian ancestry populations, we cannot determine whether the genetic affinity between Chuandong and western Yunnan populations reflects a direct connection or shared gene flow from central Yunnan and northern East Asian populations.

Overall, our results showed that the basal East Asian Xingyi_EN genetic component had disappeared in southwestern East Asia by the Bronze Age. However, the current data cannot pinpoint the specific source population that drove this replacement. Gene flow from neighboring Yunnan regions or more distant northern East Asian sources represents a plausible explanation.

### Genetic similarities of historical Guizhou and Guangxi populations

Sampling from 1100 to 300 years ago, we then explored the genetic characteristics of Guizhou populations during historical times. In the PCA plot, historical Guizhou populations overlapped with the Guangxi populations of a similar period, especially with the 500-year-old GaoHuaHua population from Guangxi (Fig. 2A). In the f3-outgroup heatmap, historical Guizhou populations and GaoHuaHua also clustered together (Fig. 2B). Statistical evaluation of this genetic relationship using D-statistics showed the historical Guangxi populations were closer to historical Guizhou populations than other northern and southern ancient East Asians (Table S4), and qpAdm modeling also supports historical Guizhou and Guangxi populations sharing ancestry (Fig. 4, Table S7).

**Figure 5.**
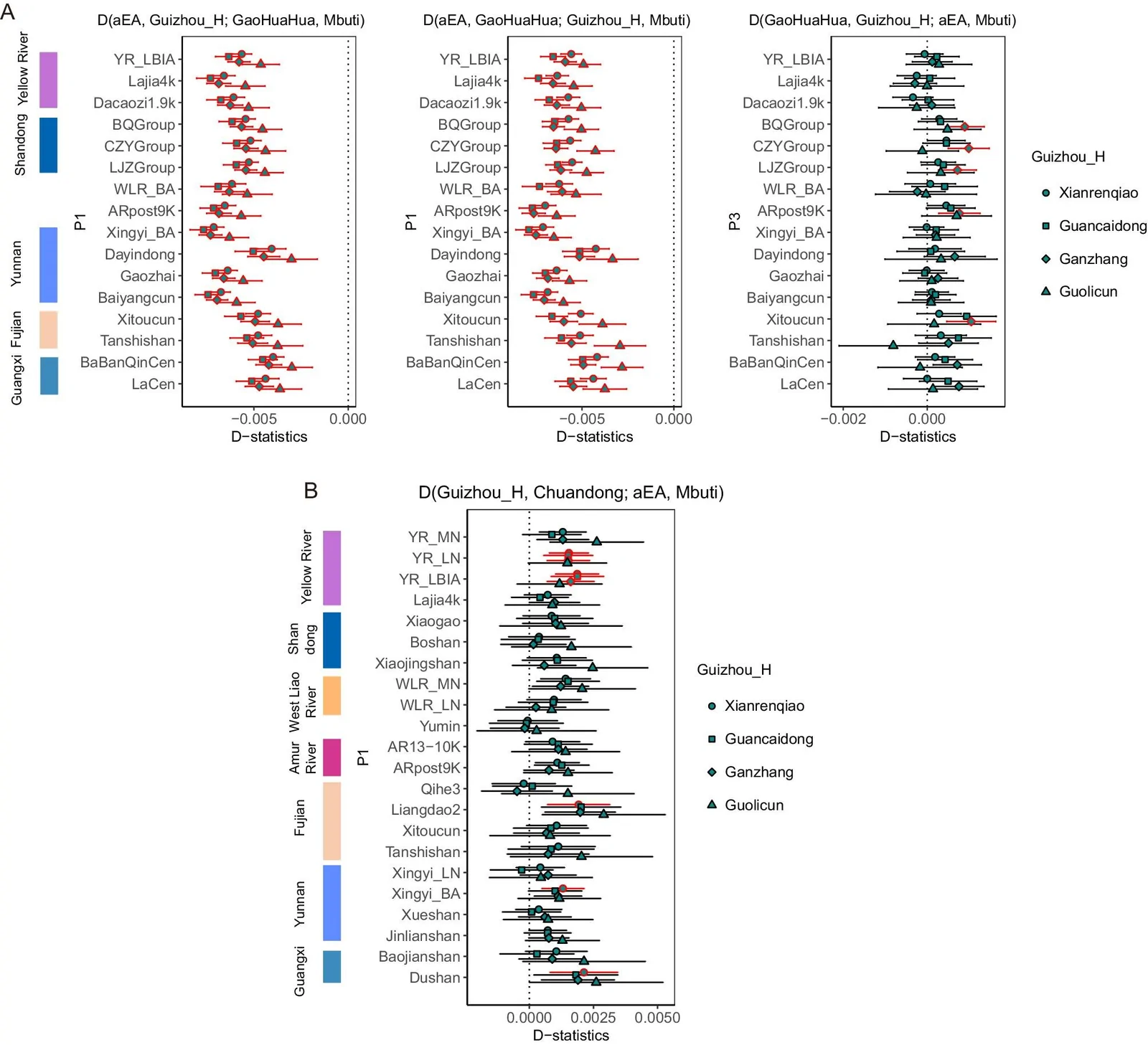
Genetic profile of historical Guizhou populations. (A) D-statistics evaluating relationships between the Bronze Age East Asians, the Historical Guizhou populations, and the 500-year-old GaoHuaHua from Guangxi. (B) Comparing Historical Guizhou populations and Chuandong with the ancient East Asians. For both (A) and (B), error bars represent 2 standard errors, and D-statistics showing |*Z*| > 2.5 are highlighted with a red outline and error bar. The sample ID, age information, and detailed geographic location of published ancient samples used in analysis can be found in Table S11.

According to records and inscriptions, the cave burial sites in the Guizhou region, including Guancaidong, Xianrenqiao, Ganzhang, and Guolicun, belonged to the ancestors of the Miao–Yao people. Previous studies have shown that among cave burial populations in Guangxi, individuals associated with Miao–Yao (Hmong–Mien) speakers, represented by the GaoHuaHua population, exhibit significant genetic differentiation from those associated with Zhuang (Tai-Kadai) speakers (for example, BaBanQinCen and Lacen populations), indicating substantial population structure by at least 500 years ago [16]. By comparing newly sequenced Guizhou historical individuals with these Guangxi populations with D-statistics, we found that the historical cave burial populations of Guizhou cluster more closely with GaoHuaHua than with other historical Guangxi sites (for example, BaBanQinCen and LaCen) and other East Asian samples (*D(aEA, Guizhou_H; GaoHuaHua, Mbuti), −*26.9 *< Z < −*4.4; *D(aEA, GaoHuaHua; Guizhou_H, Mbuti), −*25.0 *< Z < −*4.2; *D(GaoHuaHua, Guizhou_H; aEA, Mbuti), −*1.4 <* Z <* 4.1; Fig. 5A). Taken together, the historical populations from Guizhou and Guangxi affiliated with Miao–Yao ancestry show closer genetic affinity with each other than with nearby Zhuang-related populations, further supporting the conclusion that genetic differentiation between Miao–Yao and Zhuang language groups was already established at least 500 years before present.

To further investigate the migration events that shaped the genetic characteristics of historical Guizhou, we compared the historical Guizhou populations with the Neolithic Chuandong population. We found that influences of northern East Asian ancestry, as seen in the Late Neolithic and Bronze Age, also occurred in historical Guizhou (Fig. 5B). Among the Neolithic northern East Asian populations from different areas, Neolithic Yellow River ancestry gave the most significant signals of allele sharing with historical Guizhou populations, indicating it as a likely ancestry source for the gene flow during this period. Additionally, historical Guizhou populations also showed genetic affinity with the 9000-year-old Dushan individual from Guangxi, which is geographically adjacent to Guizhou (Fig. 5B). Overall, under the dual influence of the northern East Asians and more local populations, the genetic composition of the Guizhou and Guangxi populations gradually converged in the historical period.

## DISCUSSION

Our study explored the population genetic history of the Guizhou region of southwestern East Asia spanning the past 12 000 years. Our results showed that Paleolithic to Early Neolithic southwestern East Asian populations had complex genetic profiles. Guizhou, like the contemporary Guangxi and Yunnan regions, also exhibited local basal East Asian Xingyi_EN ancestry in the Paleolithic, and with significantly stronger northern East Asian genetic affinities. During the Late Neolithic and Bronze Age (4000–2000 BP), the Central Yunnan and northern East Asian ancestry continued to influence southwestern East Asia, and basal East Asian Xingyi_EN ancestry is no longer detectable in southwestern East Asia by the Bronze Age. Additional gene flow from Neolithic Guangxi- and coastal Fujian-related populations in the historical period (1100–300 BP) contributed to the genetic homogeneity between Guizhou and Guangxi populations.

Previously, the only genomic data older than 10 000 years from southern East Asia was from the 11 000-year-old Longlin individual, who primarily carried basal Xingyi_EN and genetic components similar to those in coastal Fujian populations, and the 12 000-year-old Qihe3 individual in coastal Fujian [5,16]. The current work provides genome-wide data from 5 individuals at 3 archaeological sites in Guizhou dating to before 10 000 BP, substantially expanding the genetic record of pre-Neolithic southern East Asia. Northern genetic components were previously reported to have begun appearing in coastal Fujian regions around 8300 years ago [17]; however, the newly sequenced Paleolithic genome data from Guizhou now provide evidence for regionally heterogeneous distribution patterns of northern East Asian ancestry within southwestern East Asia before 11 000 years ago, predating proposed agriculture-related Sino-Tibetan language population expansions [25–27]. Taken together, these findings suggested that either the north-to-south East Asian gene flow occurred earlier than previously reported, or that lineages related to northern East Asian populations existed in southwestern East Asia during the Paleolithic. At the end of the Pleistocene, the lithic industries in Guizhou were primarily divided into two types: the ‘Maomaodong Cave’ type, characterized by the ‘smashing technique’, and the ‘Caohai’ or ‘Ma’anshan’ type, distinguished by ‘small flake tools’ [28–30]. The discovery of a small flake tool tradition in Guizhou was believed to be related to the migrations of northern foragers to the south [31], while alternative hypotheses proposed a local origin for this technology [32]. Our new genetic results reveal that ancient individuals associated with the Small Flake Tool Tradition—such as those from the Zhaoguo and Dadong sites—carry genetic components related to northern East Asians. These findings provide, for the first time, genetic evidence supporting the hypothesis that the Small Flake Tool Tradition in Guizhou during the Paleolithic period was introduced by northern groups.

The Guizhou region is characterized by its vast karst terrain. This mountainous environment likely acted as a geographic barrier to the influx of external populations and, by offering limited land suitable for agriculture, may have made it less attractive to Neolithic farming groups. Archaeological evidence indicates less developed agricultural practices in Neolithic Guizhou than in the contemporaneous Yunnan region [33,34]. However, despite its mountainous terrain, which may limit external demographic influences, basal East Asian Xingyi_EN ancestry did not persist there into the Bronze Age, indicating that the transition from the Late Neolithic to the Bronze Age period in this region was marked by a significant population change.

Beginning at least 1100 years ago, genetic interactions between ancient populations in Guizhou and neighboring Guangxi regions intensified, and coastal Fujian population-related ancestries began appearing in Guizhou. Historical records show that toward the end of the Northern Song Dynasty, the Song government began using Guizhou as a transit hub for horse trading with Guangxi and the Dali Kingdom [35,36]. The flourishing horse trade in Guizhou and Guangxi likely facilitated population migrations and interactions between these two regions. The Han culture population at the Songshan site in Guizhou was shown to contain primarily Yellow River-associated ancestry, but was distinct from that of contemporaneous Guangxi populations [37,38]. In contrast, our newly sequenced Miao–Yao cave burial populations from Guizhou clustered with the historical Miao–Yao rather than the Zhuang cave burial populations from Guangxi. These patterns indicated that the genetic separation between Hmong–Mien speakers (Miao–Yao people) and Kra–Dai speakers (Zhuang people) was maintained within the Guizhou and Guangxi populations. Although genetic studies of present-day Southeast Asians suggested geographic isolation to be the primary driver of genetic differentiation among different linguistic groups [39], our new results indicated that linguistic and cultural distinctions may also have played significant roles in shaping genetic stratification of historical populations in southwestern East Asia.

## CONCLUSIONS

Overall, this study examines the genetic history of ancient populations in Guizhou over the last 12 000 years and provides new insights into several key questions. Our findings reveal the co-existence of northern East Asian ancestry and basal East Asian Xingyi_EN lineage in southwestern East Asia as early as 12 000 years ago. Furthermore, we provide the first genetic evidence linking the Small Flake Tool Tradition in Guizhou to the southward migration of northern hunter-gatherers. By the Bronze Age (4000–2000 BP), the Xingyi_EN-related ancestry was replaced and was no longer detected in southwestern East Asia. During the historical period, interactions between Guizhou and Guangxi populations intensified but remained constrained by language-specific affiliations: genetic differentiation between Miao–Yao and Zhuang groups was already present in this region since at least 500 years ago.

## MATERIALS AND METHODS

All wet laboratory work and data analysis are performed with equipment from the Molecular Paleontology Laboratory, Institute of Vertebrate Paleontology and Paleoanthropology, Chinese Academy of Sciences. All sampling of the specimens was approved by the relevant co-authors, archaeological institutes, universities, and the review board at the Institute of Vertebrate Paleontology and Paleoanthropology, Chinese Academy of Sciences (Review No. 202209020 012).

Detailed information about materials and methods is described in Supplementary Data at NSR online.

## Supplementary Material

nwag398_Supplemental_Files

## Data Availability

The raw sequence reads and aligned BAM files reported in this paper have been deposited in the Genome Sequence Archive (https://ngdc.cncb.ac.cn/gsa) in National Genomics Data Center (https://ngdc.cncb.ac.cn), China National Center for Bioinformation/Beijing Institute of Genomics, Chinese Academy of Sciences [40,41] (GSA-Human: PRJCA044389) that are publicly accessible at https://ngdc.cncb.ac.cn/gsa-human. The pseudo-diploid genotype calls (Eigenstrat format) data reported in this paper have been deposited in the OMIX, China National Center for Bioinformation/Beijing Institute of Genomics, Chinese Academy of Sciences [40,42] (https://ngdc.cncb.ac.cn/omix: accession no. OMIX011378).
