# Supplementary material for: Paleogenomics retraces the ancient population history of Southwestern East Asia for the past 12 000 years
Source: Natl Sci Rev. 2026 Jul 2;13(17):nwag398. doi: 10.1093/nsr/nwag398 (PMC13587099; doi:10.1093/nsr/nwag398)
Supplement: Supplementary file 1 [file nwag398_supplemental_files.zip › SupplementaryData.docx]

**MATERIALS AND METHODS**

**Ethics statement**

All sampling of the specimens was approved by the relevant co-authors, archaeological institutes, universities, and the review board at the Institute of Vertebrate Paleontology and Paleoanthropology, Chinese Academy of Sciences (Review No. 202209020012).

**Archeological information of newly sequenced samples**

This study analyzed skeletal remains from 36 ancient individuals excavated from 8 archaeological sites in Guizhou, Southwest China. The related archeological information is given below:

i. *Niupodong Site (n = 2)*

The Niupodong site is situated on a hill named Niupo, approximately 0.2 km east of Pingzhai Village, Machang Town, Gui'an New District, Guizhou Province. This archaeological site preserves five cultural phases spanning from the Late Paleolithic (~15,000 years BP) to the Spring and Autumn Period[1]. This site comprises three caves (A, B, and C), and our study sequenced the individuals M8 and M9 from Cave A. The calibrated radiocarbon dating of the M9 individual yielded a range of 11,688–11,264 cal BP (Table S1, Table S8).

ii. *Dadong Site (n = 2)*

The Qingshuiyuan Dadong site is located in Huishui County, Baijin Town, Guizhou Province. The stone products unearthed from this site are primarily from a small-scale stone flake industry, and the raw materials are mainly sourced from flint in the surrounding rock [2]. Our study sequenced two individuals from the same layer, and the calibrated radiocarbon dating of the ①SYDD-2232 individual yielded a range of 11,201–11,079 cal BP (Table S1, Table S8).

iii. *Zhaoguo Site (n = 3)*

The Zhaoguo Cave site is a large-scale cave deposit located in Yankong Village, Gaofeng Town, Gui'an New District, Guizhou Province. The site features continuous and well-preserved cultural deposits spanning approximately 40,000 to 200 years BP, encompassing the entire Late Paleolithic through Neolithic periods[3]. Our study sequenced burials M1, M2, and M7, which is genetically identical to M1, from a stratigraphic layer dated to 11,000-10,000 BP (Table S1, Table S8).

iv. *Chuandong Site (n = 5)*

The Chuandong site is in a karst peak in the west of Puding County, Anshun City, Guizhou Province. Several human fossils, a large number of stone products, animal bone, and horn products were unearthed at the site[4]. Our study sequenced 5 individuals from the layer dating to 2,000-4,000 BP (Table S1, Table S8)

v. *Guancaidong Site (n = 3) and Xianrenqiao Site (n = 13)*

Both the Guancaidong and the Xianrenqiao Cave are cave burial sites located in Shitouzhai Village, Baijin Town, Huishui County, Guizhou Province. The Guancaidong is located about 300 meters east of the Xianrenqiao Cave. These two caves are the ancestral tombs of the local Miao people with the surname Zhao in Shitouzhai village. According to historical records, the ancestors of the Zhao surname Miao people migrated from Liuzhou, Guangxi, during the Tang Dynasty and were initially buried in Guancaidong. However, due to a fire in Guancaidong, they were later reburied in the Xianrenqiao Cave. [5] Radiocarbon dating of the two remains in Guancaidong revealed that one was 1,110 ± 80 BP, and the other was 985 ± 72 BP, approximately at the transition from the Tang to the Song dynasties. [6] Based on the burial objects and coffin type found in the Xianrenqiao Cave, it is speculated that the site was used around the Ming Dynasty. [5] The calibrated radiocarbon dating of individual 14 from Xianrenqiao Cave was 646–548 cal BP (Table S1, Table S8).

vi. *Ganzhang Site (n = 7)*

The Ganzhang cave burial site is situated in Wuzhai Village, Gaopo Town, Huaxi District, Guiyang City, Guizhou Province. It is the ancestral tomb of the local Miao people and was used from the mid-Ming Dynasty to the early Qing Dynasty. Our study sequenced 7 samples at this site. The calibrated radiocarbon dating of individuals 1 and 2was 544–509 cal BP and 504–330 cal BP (Table S1, Table S8).

vii. *Guolicun Site (n = 1)*

The Guolicun cave burial site is situated in Guoli village, Wantanhe town, Longli County, Qiannan Bouyei and Miao Autonomous Prefecture, Guizhou Province. It is the ancestral tomb of the local Miao residents and was used from the mid-Ming Dynasty to the early Qing Dynasty. Our study sequenced 1 individual at this site (Table S1)

**Ancient DNA extraction, library preparation, and sequencing**

Bone powder was prepared from 36 human skeletal remains using a drilling tool with single-use drill bits to minimize contamination. The inner ear region of the petrous bone was preferentially sampled. Ancient DNA extraction and library preparation[7] were performed in the clean room of the Key Laboratory of Vertebrate Evolution and Human Origins of the Chinese Academy of Sciences, IVPP, CAS. Optimized extraction protocols and a single-stranded (SS) library preparation strategy [8,9] were employed. Three specimens from the Niupodong site and one specimen from the Chuandong site underwent uracil-DNA-glycosylase treatment (“UDG”). All other libraries were prepared without uracil-DNA-glycosylase treatment (“no UDG”) (Table S1). The complete mitochondrial genome[10] and nuclear 1.2 million SNPs (the “1240k” dataset as described in [11–13]) were captured using a hybridization oligonucleotide probe (Table S1). Enriched mtDNA libraries were sequenced on Illumina MiSeq platforms (2×76 bp paired-end reads), and nuclear DNA libraries were sequenced on either Illumina NovaSeq X Plus or HiSeq Xten platforms (2×150 bp paired-end reads).

**Bioinformatics post-processing and contamination assessment**

Adapter trimming and read merging were performed using leeHom[14], with overlapping forward and reverse sequences (minimum 11 bp overlap) merged into single reads. Processed reads were aligned to the human reference genome (hg19) using BWA[15] (v0.5.10-evan.10-1-gfb1ff83,bwa bam2bamcommand) with parameters “-n 0.01 -l 16500 -o 2” for “no UDG” libraries and default parameters for “UDG” libraries. Reads with mapping quality (MAPQ) <30 were discarded, and PCR duplicates (defined as reads sharing identical orientation, start, and end positions) were removed, retaining only the highest-quality read per duplicate set. The C-to-T substitution rate at terminal nucleotides was calculated for each individual as evidence of ancient DNA authenticity (Table S1). Mitochondrial contamination was estimated for each individual using ContamMix [10,16]. For male individuals, the X-chromosome contamination was calculated using ANGSD[17].

**Pseudo-haploid genotype calling and quality control**

For all single-stranded (SS) “no UDG” libraries, both the C/T bases in forward-mapping reads and the G/A bases in reverse-mapping reads were masked within the 5 terminal nucleotides at both ends of the merged read to minimize the impact of cytosine deamination. For other single-stranded (SS) “UDG” libraries, the same masking strategy was adopted for the terminal nucleotide at the 5’ end and the 2 terminal nucleotides at the 3’ end. For two Niupodong individuals and one Chuandong individual, both “UDG” and “no UDG” libraries were masked separately and then merged. The pseudo-haploid genotypes for each individual were determined by randomly selecting one masked fragment per genomic position using pileupCaller (https://github.com/stschiff/sequenceTools). For individuals with mitochondrial or X-chromosome contamination levels greater than 5%, only fragments with ancient DNA damage patterns were masked and genotyped (Table S1). Individuals with < 15,000 successfully genotyped SNPs were excluded, retaining 32 individuals for downstream analyses (Table S1).

**Sex Determination and Uniparental Haplogroup Assignment**

Biological sex was determined genetically by quantifying the ratio of mapped Y-chromosome reads to autosomal and X-chromosome reads, considering only reads with a mapping quality (MAPQ) of≥37 [18]. For mitochondrial haplogroup assignment, consensus sequences were generated using reads of 30 bp or longer with a MAPQ score of 30 or higher. Haplogroup calls were made using Haplogrep2[19] based on Phylotree Build 17[20]. For male individuals, Y-chromosome haplogroups were determined by phylogenetic analysis using the International Society of Genetic Genealogy dataset, applying quality filters (MAPQ ≥30, base quality ≥30). The most derived Y-chromosome SNP assignment required at least two derived SNPs, if the bases represented C-to-T or G-to-A variants, [21], for all sex assignments and haplogroups. Results are provided in Table S1.

**Eliminating kinship bias**

The kinship relatedness among Guizhou individuals was estimated using READ_v1 software[22]. This method evaluates allele sharing patterns through the proportion of non-matching alleles (P0) to classify relationships into distinct categories (unrelated, first-degree, second-degree, or identical). This kinship analysis identified 28 genetically unrelated individuals for subsequent population genetics studies, achieved by excluding close relatives while retaining the specimen with the highest SNP coverage within the 5 kinship groups (Table S1).

**Dataset compilation**

The published 1240k SNP genotype dataset of ancient and present-day East Asians and Eurasians was selected from the Allen Ancient DNA Resource (AADR_v62.0) database[23]. This dataset was then merged with recently published East Asian samples from Yunnan and Shandong [24,25] and the newly sequenced Guizhou individuals. The sample IDs, age information, and geographic locations of the published samples used in our analysis are described in Table S11.

**Principal components analysis**

Principal component analysis (PCA) was conducted using the smartpca program[26] from the EIGENSOFT package with default parameters, except for enabling the lsqproject: YES option for the projection of ancient samples. The reference PC space was constructed using modern Eurasian populations. Ancient samples (both newly sequenced and previously published) were subsequently projected onto this reference space. The resulting PCA plot, including comprehensive labeling of all modern reference populations, is presented in Figure S1.

**Outgroup-f3 and D-statistics**

The pairwise outgroup-*f3* statistics were computed using qp3Pop (v412) from the ADMIXTOOLS package[27] with the ‘Mbuti’ modern hunter-gatherers from Central Africa as the outgroup. The results were visualized as a heatmap using the R package *heatmap.2* (<https://search.r-project.org/CRAN/refmans/gplots/html/heatmap.2.html>). The D-statistics were calculated using qpDstat (v712) from the ADMIXTOOLS package[27] and in the form of *D(P1, P2; P3, Mbuti)*.

**Admixture modeling with qpAdm**

The genetic ancestry components of Guizhou populations were estimated using qpAdm (v634) from the ADMIXTOOLS package [27] with a systematic rotating scheme to identify the optimal admixture models. The qpAdm analysis was conducted with the "allsnps: YES" parameter to eliminate the influence of the high missing data rate of several individuals.

A ‘basal’ set of reference populations, including Mbuti, Okunevo_EMBA, Mota, UstIshim, Kostenki14, Ganj_Dareh_N, Yana, Karelia, Clovis, LBK_EN, Kotias, Shamanka_EN, IndusPeriphery (merged Gonur2_BA and Shahr_I_Sokhta_BA2), and Kolyma, was applied for all qpAdm analysis.

i. *Modeling the ancestries of Paleolithic Guizhou populations*

In addition to the ‘basal’ reference populations, the additional set of reference populations, including Tianyuan, AR33K, Hoabinhian (La368), JpKa6904 (Jomon8.8k), Qihe3, Liangdao2, Xingyi_EN, Bianbian, Boshan, AR19K, and AR14K, were used as ‘rotating’ sources in the qpAdm analysis of Paleolithic Guizhou populations. All these ‘rotating’ sources, except Mbuti, were assigned as reference or right populations, if they were not assigned as source or left populations. Then Longlin, ZhaoguoM1, YR_MN, and Lajia4k were used as ‘non-rotating’ source populations, as they were likely to have had gene flow to Paleolithic Guizhou populations or were much younger in age than the target populations, and were therefore not suitable as reference populations. All feasible one- and two-way qpAdm mixture models for Paleolithic Guizhou populations were presented in Table S5.

ii. *Modeling the ancestries of Later Neolithic and Bronze Age Guizhou, Yunnan, and Fujian populations*

To explore the genetic mixture components and whether the basal Asian ancestries remained, the same ‘rotating’ sources as those used for Paleolithic Guizhou populations were applied to Later Neolithic and Bronze Age populations from Guizhou, Yunnan, and Fujian. Besides the Longlin, ZhaoguoM1, YR_MN, and Lajia4k, Xingyi_LN were also used as ‘non-rotating’ source populations in the qpAdm analysis. All feasible one- and two-way qpAdm mixture models for Late Neolithic and Bronze Age populations in Guizhou, Yunnan, and Fujian were presented in Table S6.

iii. *Modeling the ancestries of Historical Guizhou populations*

Since the Historical Guizhou samples show genetic affinity to Neolithic and Historical Guangxi populations, the Neolithic Dushan and Baojianshan populations were added to the ‘rotating’ sources, and the Historical BaBanQinCen, LaCen, and GaoHuaHua were added to the ‘non-rotating’ sources in the qpAdm analysis. All feasible one- and two-way qpAdm mixture models for Historical Guizhou populations are presented in Table S7.

**Constructing the maximum likelihood phylogenetic tree with admixture events**

We applied TreeMix (version v1.13) to infer the maximum likelihood phylogenetic tree with admixture events. Trees were rooted with the Central African Mbuti population. We included ancient human groups with ancestry representative of diverse human lineages present in eastern Eurasia, including the 45,000-year-old UstIshim individual, the 40,000-year-old Tianyuan individual, the 8,000-year-old Hoabinhian from Southeast Asia (La368), the 7,100-year-old Xingi_EN individual from central Yunnan, the Amur River region population (AR14K), coastal Shandong individuals (we merged Bianbian and Xiaogao as Shandong_EN), and inland Northern East Asians (Shimao_LN and Yumin), three Tibetan Plateau populations (Zongri5.1k, Shannan3k, Chamdo2.7k), the earliest southern East Asian Qihe3 individual, and Early Neolithic populations from Fujian (we merged Qihe and Liangdao2 as Fujian_EN). The sample IDs of these published data are listed in Table S11. For the newly sequenced samples, we used the ZhaoguoM1 individual, which had the highest number of SNPs, to represent the Paleolithic Guizhou ancestry.

To decide the best value of migration numbers (m), we plotted the log likelihood score curve against the admixture edge number. Adding additional admixture edges will always improve the likelihood score, but beyond a certain value, more edges will only slightly improve it. For our data, we accepted five as the optimal number of migrations, since exceeding this value, the slope of the likelihood curve will decrease (Figure S2). We then performed 1,000 bootstrap replicates with m=5. We found that Xingyi_EN, Longlin, and ZhaoguoM1 were outgroups to other ancient northern and southern East Asians, with bootstrap support of 99.9% in the m=5 tree (Figure S4). Although our results did not support Longlin and ZhaoguoM1 forming a clade relative to Xingyi_EN (bootstrap = 25.5%), Longlin and ZhaoguoM1 still shared a migration from Xingyi_EN (Figure S4). In the trees with m=2, m=4, and m=5, we always found migration events connecting ZhaoguoM1 and Shannan3k (Figure S3). Based on D-statistics and qpAdm, we assumed that Shannan3k represents a broad Neolithic Northern East Asian ancestry rather than Tibetan ancestry. In the m=5 tree, Longlin also has a migration event into Chamdo2.7k, but its admixture weight is smaller than that of ZhaoguoM1 (Figure S4).

**Admixture graph modeling using qpGraph**

We used the ADMIXTOOLS2 R package to automatically fit the admixture graphs (AGs) to our data. We included the Central African Mbuti population, the 37,000-year-old Eurasian Kostenki14 individual, the 40,000-year-old Tianyuan individual, the 8,000-year-old Hoabinhian individual (La368), the 7,100-year-old Xingyi_EN individual, Neolithic inland and coastal Northern East Asians (Boshan and YR_MN individuals), the oldest coastal Southern Fujian individual (Qihe3), and the newly sequenced ZhaoguoM1 individual in Guizhou. The sample IDs for the published data are listed in Table S11. Since the bootstrap phylogenetic analysis could not fully resolve the relationships among Xingyi_EN, Longlin, and ZhaoguoM1 into a consensus bifurcating tree (Figure S4), we omitted Longlin from our first attempt. To avoid bias due to manually modifying and expanding the explored topology space, we applied the find_graph() function to automatically fit the admixture graphs, with only Tianyuan, Hoabinhian, and Xingyi_EN restricted to being unadmixed.

The results showed that, even in a graph with only one admixture event, ZhaguoM1 was estimated to contain 55% ancestry from the ancestral node of Boshan and YR_MN and 45% Xingyi_EN-related ancestry (Figure S5). This is consistent with our conclusion that ZhaoguoM1 was admixed between Xingyi_EN-related ancestry and an ancient broad Northern East Asian ancestry. To further investigate whether this Northern ancestry influenced the Longlin individual, we included Longlin in our second qpGraph dataset. We found that if we assumed only two admixture events, ZhaoguoM1 and Longlin were estimated to be sister groups (Figure S6A). However, when we increased the number of admixture events to three or four, the Northern East Asian influence on ZhaoguoM1 became apparent, and this ancestry did not contribute to the Longlin individual (Figure S6B-C).

**REFERENCES**

1. South China Institute of Archaeology, Chinese Academy of Social Sciences, Guizhou Provincial Institute of Cultural Relics and Archaeology, Pingba County Cultural Relics Management Office. Brief report on the excavation of Niupodong site in Pingba County, Guizhou Province, 2012-2013 [in Chinese]. Archaeology 2015;8:16-36+2.

2. Zhang X, Pei S, Wu X et al. Qingshuiyuan Dadong: A newly discovered Late Paleolithic site in Guizhou province, China. Archaeological Research in Asia 2017;:13–21.

3. Guizhou Provincial Institute of Cultural Relics and Archaeology, School of Archaeology and Museology, Sichuan University, Chengdu Cultural Relics and Archaeology Research Institute. Brief report on the excavation of Zhaoguo Cave site in Yankong Village, Gui’an New District, Guizhou, in 2020 [in Chinese]. Archaeology 2024;4:3-20+129.

4. MAO Y, WANG D, WU K et al. Characteristic and significance of prehistoric sites in Puding County [in Chinese]. Guizhou Science 2019;37:40–3.

5. Li F. The Regression of the Soul: Rock Paintings in Southwest China, Focused on the Coffin Paintings in the Rock Cave at Xianren Bridge, Huishui County, Guizhou Province [in Chinese]. Southern Ethnology and Archaeology 2012:225–72.

6. LI M. A Study of the “Coffin Cave”in Pingba of Central Guizhou [in Chinese]. Journal of Guiyang University(Social Sciences) 2021;16:82-85+107.

7. Dabney J, Knapp M, Glocke I et al. Complete mitochondrial genome sequence of a Middle Pleistocene cave bear reconstructed from ultrashort DNA fragments. Proc Natl Acad Sci USA 2013;110:15758–63.

8. Meyer M, Kircher M. Illumina sequencing library preparation for highly multiplexed target capture and sequencing. Cold Spring Harb Protoc 2010;2010:pdb.prot5448.

9. Kircher M, Sawyer S, Meyer M. Double indexing overcomes inaccuracies in multiplex sequencing on the Illumina platform. Nucleic Acids Res 2012;40:e3.

10. Fu Q, Meyer M, Gao X et al. DNA analysis of an early modern human from Tianyuan Cave, China. Proceedings of the National Academy of Sciences 2013;110:2223–7.

11. Fu Q, Hajdinjak M, Moldovan OT et al. An early modern human from Romania with a recent Neanderthal ancestor. Nature 2015;524:216–9.

12. Haak W, Lazaridis I, Patterson N et al. Massive migration from the steppe was a source for Indo-European languages in Europe. Nature 2015;522:207–11.

13. Yang MA, Gao X, Theunert C et al. 40,000-Year-Old Individual from Asia Provides Insight into Early Population Structure in Eurasia. Current Biology 2017;27:3202-3208.e9.

14. Renaud G, Stenzel U, Kelso J. leeHom: adaptor trimming and merging for Illumina sequencing reads. Nucleic Acids Res 2014;42:e141.

15. Li H, Durbin R. Fast and accurate short read alignment with Burrows-Wheeler transform. Bioinformatics 2009;25:1754–60.

16. Fu Q, Mittnik A, Johnson PLF et al. A Revised Timescale for Human Evolution Based on Ancient Mitochondrial Genomes. Current Biology 2013;23:553–9.

17. Korneliussen TS, Albrechtsen A, Nielsen R. ANGSD: Analysis of Next Generation Sequencing Data. BMC Bioinformatics 2014;15:356.

18. Skoglund P, Storå J, Götherström A et al. Accurate sex identification of ancient human remains using DNA shotgun sequencing. Journal of Archaeological Science 2013;40:4477–82.

19. Weissensteiner H, Pacher D, Kloss-Brandstätter A et al. HaploGrep 2: mitochondrial haplogroup classification in the era of high-throughput sequencing. Nucleic Acids Res 2016;44:W58–63.

20. van Oven M, Kayser M. Updated comprehensive phylogenetic tree of global human mitochondrial DNA variation. Human Mutation 2009;30:E386–94.

21. Wang H, Yang MA, Wangdue S et al. Human genetic history on the Tibetan Plateau in the past 5100 years. Science Advances 2023;9:eadd5582.

22. Kuhn JMM, Jakobsson M, Günther T. Estimating genetic kin relationships in prehistoric populations. PLOS ONE 2018;13:e0195491.

23. Mallick S, Micco A, Mah M et al. The Allen Ancient DNA Resource (AADR) a curated compendium of ancient human genomes. Sci Data 2024;11:182.

24. Wang T, Yang MA, Zhu Z et al. Prehistoric genomes from Yunnan reveal ancestry related to Tibetans and Austroasiatic speakers. Science 2025;388:eadq9792.

25. Liu J, Liu Y, Zhao Y et al. East Asian Gene flow bridged by northern coastal populations over past 6000 years. Nat Commun 2025;16:1322.

26. Patterson N, Price AL, Reich D. Population Structure and Eigenanalysis. PLoS Genetics 2006;2:e190.

27. Patterson N, Moorjani P, Luo Y et al. Ancient Admixture in Human History. Genetics 2012;192:1065–93.
