# Supplementary material for: Paleogenomics retraces the ancient population history of Southwestern East Asia for the past 12 000 years
Source: Natl Sci Rev. 2026 Jul 2;13(17):nwag398. doi: 10.1093/nsr/nwag398 (PMC13587099; doi:10.1093/nsr/nwag398)
Supplement: Supplementary file 1 [file nwag398_supplemental_files.zip › SupplementaryFigure.docx]

**Figure S1. The full-scale (A) and room in (B) principal component analysis (PCA) plot displaying genetic relationships among newly sequenced Guizhou individuals and ancient/modern populations.** All ancient individuals are projected onto the principal component space defined by modern reference populations. Newly sequenced Guizhou samples are denoted by solid symbols for visual distinction. Yellow points are Paleolithic Guizhou samples, purple/blue points are Late Neolithic/Bronze Age Guizhou samples, and green points are historical Guizhou samples. The PCA plot region of the room in (B) plot is indicated by black dotted line square in the full-scale (A) plot. Related to Figure 2.

**Figure S2: The curve of the Treemix model likelihood value and the number of admixture edges.** The ln(likelihood) value increases with the admixture number, but beyond a certain value, the improvement decreases. Here, we choose the optimal value of the migration numbers (m) as 5.

**Figure S3: Treemix models with 0 to 5 migration events.** The maximum-likelihood phylogenetic trees were constructed with migration events among diverse representative ancient East Asian populations. The m=5 tree is replicated with bootstrap in Figure S4.

**Figure S4: Maximum likelihood tree with five migration events showing the genetic relationships of ancient humans from East Asia.** The bootstrap value for 1000 replicates is added in each node.

**Figure S5: The admixture graph illustrating the genetic relationship between ZhaoguoM1 and other ancient East Asians.** This model best fit the observed *f*-statistics under the assumption of one admixture event and restricting Tianyuan, La368, and Xingyi_EN to being unadmixed.

**Figure S6: The admixture graph incorporating Longlin.** (A) The best-fitting model with two admixture events. (B) The best-fitting model with three admixture events. (C) The best-fitting model with four admixture events.
